# Pragmatic O-Positive Whole-blood RandoMizaTion in male trauma Patients (POWeR-MTP)

**DOI:** 10.1007/s00068-025-02848-0

**Published:** 2025-04-16

**Authors:** Anthony M. Strada, Gus Suarez, Xian Luo-Owen, Maryam B. Tabrizi, Martin G. Rosenthal, Wesley T. Stevens, Sharon S. Lum, Kaushik Mukherjee

**Affiliations:** 1 Department of Surgery, Cedars-Sinai Medical Center, Los Angeles, CA USA; 2 Loma Linda University School of Medicine, Loma Linda, CA USA; 3 Division of Acute Care Surgery, Loma Linda University Health, 11175 Campus Street, Loma Linda, CA CP 21111, 92350 USA; 4 Department of Pathology, Loma Linda University Health, Loma Linda, CA USA; 5 Division of Surgical Oncology, Loma Linda University Health, Loma Linda, CA USA

**Keywords:** Traumatic hemorrhagic shock, Low-titer O-positive whole blood, Component therapy, Massive transfusion

## Abstract

**Purpose:**

Hemorrhage is a significant cause of trauma-related death. Low-titer O-positive whole blood (LTOWB) is an alternative to component therapy (CT) [packed red blood cells (PRBC) and fresh frozen plasma (FFP)]. We evaluated if LTOWB reduces transfusion requirement or mortality.

**Methods:**

Adult male trauma activations requiring uncrossmatched transfusion in the emergency department underwent nonblinded 24-hour block randomization to receive uncrossmatched LTOWB or CT in the emergency department (ED). Female patients, children, and known prisoners were excluded. If LTOWB was not available, CT was used. Primary outcome was transfusion requirement in patients surviving ≥ 24 h, with a subset analysis for patients undergoing hemorrhage control interventions (HCI). Dichotomous variables were evaluated with Chi-Square testing and continuous outcomes with Student’s T-test.

**Results:**

Overall, 199 patients were randomized (52 LTOWB, 147 CT); 36 patients (12 LTOWB, 24 CT) were excluded post-randomization for mortality within 24 h. The remaining 40 LTOWB and 123 CT patient cohorts had similar age, Glasgow Coma Scale, Injury Severity Score, heart rate, systolic blood pressure, and temperature. LTOWB patients received 1.4 ± 0.75 LTOWB units. LTOWB patients trended toward less transfusion (PRBC [3.8 ± 5.6 vs. 5.7 ± 6.2 units, *p* = 0.077], FFP [2.3 ± 3.8 vs. 3.5 ± 4.3 units, *p* = 0.088], and CRYO [0.13 ± 0.34 vs. 0.28 ± 0.68 units, *p* = 0.061]). Mortality was similar (LTOWB:10.2% [4/39] vs. CT:10.5% [13/123], *p* = 0.956). LTOWB patients undergoing HCI had less transfusion than CT patients (PRBC [3.9 ± 5.1 vs. 7.4 ± 7.2 units, *p* = 0.013]; in the HCI cohort the differences were even more pronounced when severe traumatic brain injury (TBI) deaths were excluded (PRBC [3.0 ± 3.6 vs. 7.4 ± 7.2 units, *p* < 0.001], FFP [2.1 ± 2.3 vs. 4.5 ± 5.2 units, *p* = 0.005]).

**Conclusion:**

LTOWB is associated with reduced PRBC transfusion in patients undergoing HCI, and a trend toward decreased PRBC, FFP, and CRYO transfusion in all patients.

**Trial registration:**

ClinicalTrials.gov (NCT05081063), posted 10/18/2021.

## Introduction

Traumatic hemorrhagic shock continues to be a leading cause of preventable mortality after major trauma [1]. The cornerstone of mortality prevention after traumatic hemorrhagic shock remains balanced blood product resuscitation coupled with rapid hemorrhage control [2–4]. When blood transfusion was incorporated into medical practice, the first transfusions performed were whole blood transfusions; this practice continued through World War I, World War II, the Korean and Vietnam Wars. By the end of the Vietnam conflict, whole blood had been separated into components for ease of use and focused transfusion [5, 6]. 

More recent investigations have revealed that patients have fewer deaths from hemorrhage when the ratios of fresh frozen plasma (FFP) to packed red blood cell (PRBC) units and platelets (PLT) to PRBC units approach 1:1. Matched components more closely approximate the ratios in whole blood [7, 8]. The reintegration of fresh whole blood, followed by cold stored whole blood, into modern military transfusion practice was initiated during recent conflicts in Iraq and Afghanistan and demonstrated improved survival compared to component therapy in military data [9–16]. As fresh whole blood is not available in the civilian trauma system, cold-stored whole blood has since been adapted for the civilian setting, with one unit considered equivalent to 1 unit of PRBC and 1 unit of FFP, with reduced platelet activity [5, 17–25]. 

Observational and retrospective studies have demonstrated potentially improved survival with whole blood [17, 22, 26]. Randomized controlled trials have been impaired by selection bias and differences between treatment groups; these studies do not reflect a mortality benefit but do, within limits, offer some reduction in transfusion with the addition of LTOWB [27, 28]. We sought to perform a pragmatic trial in which randomization did not impede the rapid delivery of care to patients in hemorrhagic shock. We hypothesized that initial use of LTOWB would decrease transfusion requirements compared to CT.

## Methods

### Study design

**Fig. 1:**
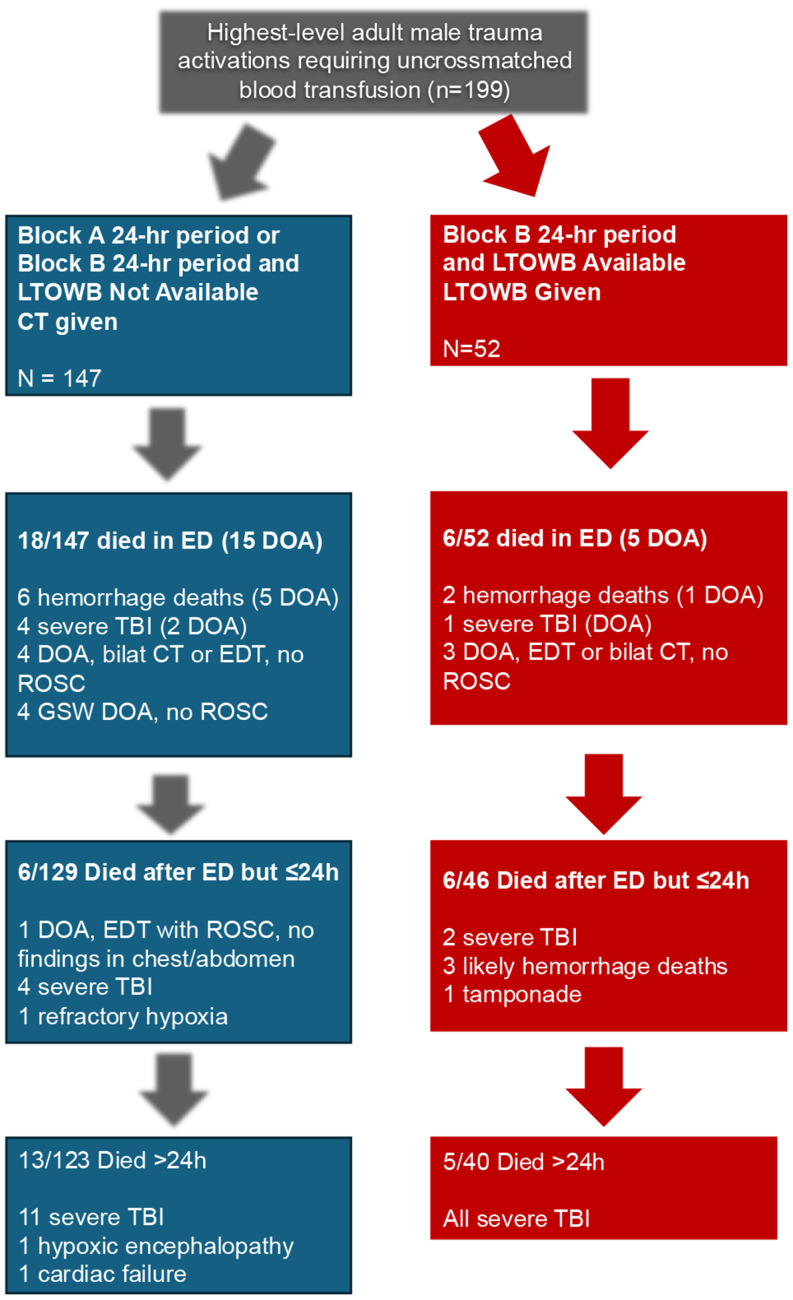
Patient Randomization and Exclusion Schema. Adult male trauma patients who were the highest-level trauma activations were enrolled at the time that uncrossmatched blood transfusion was felt to be indicated by the trauma surgeon. Blocks A and B alternated every 24 h, with CT given during Block A and LTOWB given during Block B. After the supply of LTOWB was exhausted during Block B, further transfusion was given as 1:1:1 transfusion using CT. If no LTOWB was available during Block B, CT was used. Deaths in the ED and within 24 h of arrival were specifically excluded as the primary endpoint of transfusion requirement would be affected by survivor bias if early mortalities were not excluded. The final number of patients in the two groups was imbalanced due to supply constraints of LTOWB, resulting in a significant number of patients defaulting to CT

We performed a randomized controlled superiority trial comparing LTOWB to CT as the index transfusion product at a single, American College of Surgeons Committee on Trauma-verified level 1 trauma center. All male patients over the age of 18 years who presented to the emergency department (ED) as highest-level trauma activations requiring emergency transfusion were included. Patients were randomized to transfusion with LTOWB or CT in alternating 24 h periods. Indication for uncrossmatched transfusion included a systolic blood pressure less than 90 at any time during the initial resuscitation and initiation of transfusion was required within 10 min of the onset of hypotension. Initial transfusion with CT consisting of PRBC and FFP in a 1:1 ratio versus initial transfusion of LTOWB alternated by 24 h period. During 24 h period A, all patients received CT; during 24 h period B, all patients received LTOWB. Anticipating possible supply shortages of LTOWB, if no LTOWB was available, CT was used and the patient was crossed over to the CT arm. **(**Fig. 1**)**. No crossover was permitted from CT to LTOWB. Due to the time-dependent reduction of platelet activity in LTOWB, indications for platelet transfusion were similar in each group. Intention to treat analysis was not used as the issue of LTOWB supply was outside the scope of study randomization and inherently a random, not systematic, cause of error [29, 30]. At the time of the study, our blood bank could supply two to four units at a time for the emergency department; that has since increased to eight units. During the period of this study, running out of LTOWB supply occurred approximately once or twice a week on average.

All uncrossmatched blood, including LTOWB, PRBC, and FFP, was stored in an ED blood refrigerator. LTOWB available at our center was leukoreduced but had reduced platelet activity with time. The titer cutoff for reaction against A and B cells was 1:200, with samples being classified as LTOWB if there were no reactions seen on both tubes [31–33]. Our standard of care is to use viscoelastic testing with the ROTEM device for all highest-level trauma activations. One sample is drawn at the time of arrival, with further testing at the discretion of the trauma surgeon.

Exclusion criteria for this study included children, prisoners, and female patients due to the potential risk for alloimmunization [34–37]. Patients who expired within 24 h of arrival to the emergency department were randomized but their transfusion data were excluded retrospectively from the analysis of transfusion during hospitalization to eliminate survivor bias. The rationale for this is that we would not have the statistical power to detect a difference in this small cohort of patients attributable to LTOWB. We also believed that many of the deaths occurring within the first 24 h were likely not preventable; our data is remarkably similar to previously published data from a predominantly blunt trauma population with a large geographic distribution that revealed a bimodal, not trimodal, distribution of death, with one peak in the first 24 h and the second at 24–48 h [38]. The causes of death can be seen in Fig. 1 with additional patient-level detail seen in SDC 1. Patient demographics, mechanism of injury, injury characteristics, vital signs, volume of transfusion, hemorrhage control interventions, mortality, and disposition outcomes were collected. Transfusion unit equivalents of all resuscitative products provided during the entire hospitalization from emergency department arrival to discharge or death were recorded.

The planned enrollment time was 14 months with a target patient enrollment of 200 patients prior to exclusion criteria. This enrollment goal was based on a power analysis using trial data from Cotton: accrual of 190 patients was calculated for 90% power to detect a 2-unit difference in PRBC transfusion with an alpha level of 0.05 [27]. The study was not powered to detect a mortality difference as previous studies have not been able to detect an all-cause mortality benefit between comparison groups. Ethical approval was obtained from the Institutional Review Board at the authors’ institution (IRB#5210170) with waiver of consent and the trial was listed on ClinicalTrials.gov. The waiver of consent was based on the rationale that both LTOWB and CT transfusion are acceptable community standards of care for traumatic hemorrhagic shock and no pre-intervention or community consent was required [39]. 

### Outcomes and statistical analysis

The primary outcome was volume of transfusion of LTOWB, PRBC, FFP, CRYO, and PLT units during the hospital admission. The secondary outcome was all-cause mortality. Dichotomous variables were evaluated with Chi-Square testing and continuous outcomes with Student’s T-test after verifying that distributions were normally distributed. Distributions for Glasgow Coma Scale, ventilator days, ICU length of stay, and hospital length of stay were not normally distributed, and were presented as median with interquartile range and analyzed using the Independent Samples Median Test with Yates Continuity Correction.

A subset analysis was performed for patients who received a hemorrhage control intervention (HCI). HCI included surgical (thoracotomy, sternotomy, laparotomy, and exploration of the neck, extremities, or penetrating wounds) or angioembolization procedures. Diagnostic laparoscopy to rule out bowel injury was not included as an HCI. A similar analysis was performed for patients not undergoing HCI. Within-groups analysis was performed for LTOWB and CT patients to determine the difference in transfusion requirements between HCI and non-HCI patients receiving LTOWB and HCI and non-HCI patients receiving CT. A second subset analysis was performed after also excluding all deaths due to severe traumatic brain injury, defined to be consistent with the Trauma Quality Improvement Program definition (GCS < 9 with a positive head CT).

## Results

**Fig. 2:**
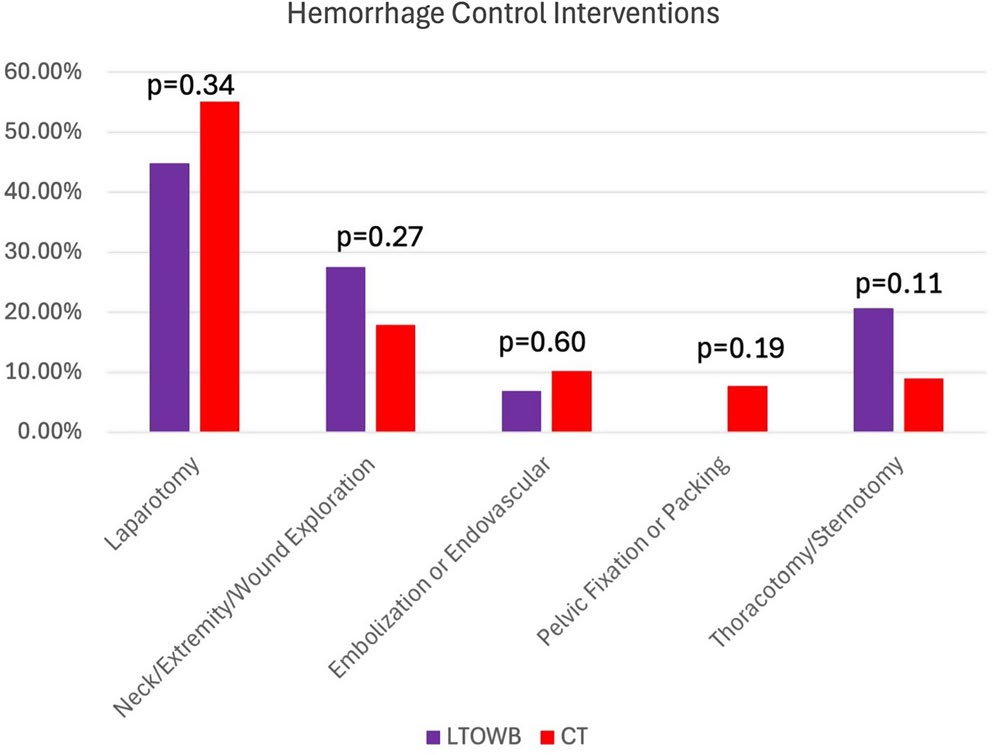
Types of Hemorrhage Control Interventions Performed (some patients received multiple interventions). The operative or interventional procedures that were performed in the CT and LTOWB groups to achieve hemorrhage control are tabulated above

Between March 1, 2022 and May 31, 2023, 199 highest-level activation patients arrived in traumatic hemorrhagic shock necessitating initiation of blood product transfusion in the emergency department. 18 patients in the CT group and 6 in the LTOWB group died in the emergency department. (Fig. 1) An additional six patients died in each group within the first 24 h. Thus, 24 patients in the CT group and 12 in the LTOWB group were retrospectively excluded. Potential causes of death are listed. (Fig. 1) Types of HCI performed are indicated in Fig. 2; these interventions were not different between groups.

Of 163 remaining patients meeting inclusion criteria, 40 were in the LTOWB and 123 were in the CT cohort. The two cohorts were well-matched with respect to age, initial Glasgow Coma Scale, Injury Severity Score, heart rate, systolic blood pressure, and temperature. The CT group had a lower temperature on arrival than the LTOWB group. (Table 1)

**Table 1:** Patient demographics *N* = 163

	LTOWB (*N* = 40) Mean ± SD	CT (*N* = 123) Mean ± SD	*P* value
Age (years)	40.7 ± 14.5	42.2 ± 17.0	0.587
ISS	20 ± 13	19 ± 11	0.632
GCS in ED	Median 14 [IQR 3, 15]	Median 14 [IQR 3, 15]	0.396*
First SBP in ED	110 ± 35.0	109 ± 29.2	0.911
First HR	103 ± 30.7	103 ± 28.1	0.983
First Temp in ED (^o^C)	36.6 ± 0.7	36.2 ± 1.4	0.051

(ISS) Injury Severity Score, (GCS) Glasgow Coma Scale, (SBP) systolic blood pressure, (HR) heart rate, (ED) emergency department

*Independent Samples Median Test with Yates Continuity Correction

LTOWB patients received 1.4 ± 0.75 units LTOWB. Compared with CT patients, LTOWB patients had a trend toward reduced transfusion requirements during their total hospital stay (PRBC [3.8 ± 5.6 vs. 5.7 ± 6.2 units, *p* = 0.077], FFP [2.3 ± 3.8 vs. 3.5 ± 4.3 units, *p* = 0.088], PLT (0.43 ± 0.84 vs. 0.68 ± 1.3 units), and CRYO [0.13 ± 0.34 vs. 0.28 ± 0.68 units, *p* = 0.061]). (Table 2A)

LTOWB patients (*n* = 16) not undergoing HCI received 1.4 ± 0.81 units LTOWB but compared with CT patients (*N* = 60) had less CRYO transfusion (0 vs. 0.22 ± 0.59 units, *p* = 0.006). Transfusion of PRBC, FFP, and PLT was not appreciably different.(Table 2B).

LTOWB patients (*n* = 24) undergoing HCI received 1.5 ± 0.78 units LTOWB but compared with CT patients (*n* = 63) had less PRBC transfusion overall (PRBC [3.9 ± 5.1 vs. 7.4 ± 7.2 units, *p* = 0.013]. Transfusion of FFP, PLT, and CRYO was not appreciably different. (Table 2C)

When the category undergoing HCI had deaths from severe traumatic brain injury excluded, LTOWB patients (*n* = 20) received 1.4 ± 0.75 units LTOWB but compared with CT patients (*n* = 62) had less transfusion overall (PRBC [3.0 ± 3.6 vs. 7.4 ± 7.2 units, *p* < 0.001], FFP [2.1 ± 2.3 vs. 4.5 ± 5.2 units, *p* = 0.005]). Transfusion of PLT and CRYO was not appreciably different. (Table 2D and 3)

**Table 2:** Hospital transfusion (mean ± SD units)

A. All Patients	LTOWB(*N* = 40)	CT(*N* = 123)	*P* value
LTOWB (units)	1.4 ± 0.75	0	**< 0.001**
PRBC (units)	3.8 ± 5.6	5.7 ± 6.2	0.077
FFP (units)	2.3 ± 3.8	3.5 ± 4.3	0.088
PLT (units)	0.43 ± 0.84	0.68 ± 1.3	0.154
CRYO (units)	0.13 ± 0.34	0.28 ± 0.68	0.061
B. **Non-HCI Patients**	LTOWB (*N* = 16)	CT (*N* = 60)	P value
LTOWB (units)	1.4 ± 0.81	0	**< 0.001**
PRBC (units)	3.8 ± 6.4	3.9 ± 4.3	0.952
FFP (units)	1.1 ± 2.2	2.4 ± 2.8	0.053
PLT (units)	0.19 ± 0.54	0.42 ± 0.93	0.202
CRYO (units)	0	0.22 ± 0.59	**0.006**
C. **HCI Patients**	LTOWB (*N* = 24)	CT (*N* = 63)	P value
LTOWB (units)	1.5 ± 0.78	0	**< 0.001**
PRBC (units)	3.9 ± 5.1	7.4 ± 7.2	**0.013**
FFP (units)	3.1 ± 4.5	4.6 ± 5.1	0.190
PLT (units)	0.58 ± 0.97	0.92 ± 1.5	0.229
CRYO (units)	0.21 ± 0.42	0.33 ± 0.76	0.332
D. **HCI Patients Excluding Severe TBI**	LTOWB (*N* = 20)	CT (*N* = 62)	P value
LTOWB (units)	1.4 ± 0.75	0	**< 0.001**
PRBC (units)	3.0 ± 3.6	7.4 ± 7.2	**< 0.001**
FFP (units)	2.1 ± 2.3	4.5 ± 5.2	**0.005**
PLT (units)	0.45 ± 0.83	0.90 ± 1.5	0.097
CRYO (units)	0.15 ± 0.37	0.34 ± 0.77	0.143

(HCI) hemorrhage control interventions, (LTOWB) low titer type O Positive whole blood, (PRBC) packed red blood cells, (FFP) fresh frozen plasma, (PLT) platelets, (CRYO) cryoprecipitate

**Table 3:** Within-groups analysis

A. CT Patients	Without HCI (*N* = 60)	With HCI (*N* = 63)	*P* value
PRBC (units)	3.9 ± 4.3	7.4 ± 7.2	**0.001**
FFP (units)	2.4 ± 2.8	4.6 ± 5.1	**0.005**
PLT (units)	0.42 ± 0.93	0.92 ± 1.5	**0.032**
CRYO (units)	0.22 ± 0.59	0.33 ± 0.76	0.360
B. **LTOWB Patients**	Without HCI (*N* = 16)	With HCI (*N* = 24)	P value
LTOWB (units)	1.4 ± 0.81	1.5 ± 0.72	0.741
PRBC (units)	3.8 ± 6.4	3.9 ± 5.1	0.974
FFP (units)	1.1 ± 2.2	3.1 ± 4.5	0.067
PLT (units)	0.19 ± 0.54	0.58 ± 0.97	0.109
CRYO (units)	0	0.21 ± 0.42	**0.022**

Within the CT group, HCI patients (*n* = 63) had higher transfusion requirements (PRBC [7.4 ± 7.2 vs. 3.9 ± 4.3 units, *p* = 0.001], FFP [4.6 ± 5.1 vs. 2.4 ± 2.8 units, *p* = 0.005], PLT [0.33 ± 0.76 vs. 0.22 ± 0.59 units, *p* = 0.032]). CYRO was not appreciably different. Within the LTOWB group, however, only CRYO was higher in the HCI group (0.21 ± 0.42 vs. 0, *p* = 0.022).

There were no significant differences in ICU length of stay, hospital length of stay, days of ventilator support, or all-cause mortality between groups. (Table 4)

**Table 4:** Mortality and hospital outcomes

	LTOWB (*N* = 40) (Median [IQR])	CT (*N* = 123) Median [IQR])	*P* value
ICU LOS (days)	2.0 [0.0, 4.0]	2.0 [0.0, 5.0]	0.765
Ventilator Duration (days)	1.0 [0.0, 3.0]	1.0 [0.0, 3.0]	0.745
Hospital LOS (days)	6.5 [1.0, 14.8]	7.0 [2.0, 18.2]	0.993
Overall Mortality (%)	10.2 (4/39)	10.5 (13/123)	0.956

(ICU) intensive care unit, (LOS) length of stay

## Discussion

We conducted a pragmatic, randomized controlled trial to assess the efficacy of LTOWB as the index transfusion product in traumatically injured patients with hemorrhagic shock. This study demonstrated that LTOWB is associated with a trend toward reduced transfusion requirements, specifically PRBC, FFP, and CRYO, in adult male trauma patients requiring emergency uncrossmatched blood transfusion, although there was no difference in all-cause mortality.

A subset analysis of patients undergoing HCI indicated increased potential RBC savings in this group of patients. The LTOWB group had 3.9 ± 5.1 units PRBC transfused as opposed to 7.4 ± 7.2 units PRBC transfused in the CT group (*p* = 0.013). Thus, for a mean of 1.5 units LTOWB transfused, 3.5 units PRBC were saved, or 2.3 units PRBC saved per unit of LTOWB transfused. In patients not undergoing HCI, there was a small but statistically significant reduction in CRYO transfusion (0 vs. 0.22 ± 0.59 units, *p* = 0.006) associated with LTOWB transfusion of 1.4 ± 0.81 units. Savings were even more pronounced if deaths due to severe traumatic brain injury were excluded. The CRYO findings were somewhat unexpected as neither LTOWB nor FFP units are cryo-reduced and thus would not be expected to function differently in this regard; at our institution a CRYO 10-pack is provided with the third massive transfusion cooler and every other cooler thereafter.

Results of the within-groups comparison are also quite illustrative. In patients receiving LTOWB, patients undergoing HCI had no significant difference in the amount of LTOWB, PRBC, or PLT transfusion compared to non-HCI patients. The HCI group receiving LTOWB had a trend toward increased FFP use (3.1 ± 4.5 vs. 1.1 ± 2.2 units, *p* = 0.061) and did use more CRYO (0.21 ± 0.42 vs. 0 units, *p* = 0.022). However, CT patients undergoing HCI required significantly more PRBC (7.4 ± 7.2 vs. 3.9 ± 4.3 units, *p* = 0.001), FFP (4.6 ± 5.1 vs. 2.4 ± 2.8 units, *p* = 0.005), and PLT (0.92 ± 1.5 vs. 0.42 ± 0.94 units, *p* = 0.032). As the types of HCI in each group were similar (Fig. 2), this is difficult to explain in the absence of a treatment effect associated with LTOWB transfusion.

Preventable mortality from hemorrhagic shock has been the focus of trauma research across battlefield and civilian settings [1, 40]. While continued efforts aim to optimize care from the prehospital setting through time of discharge, timely blood product resuscitation remains the foundation of success [1, 7, 41]. Refinement of transfusion formulas has proven the benefit of massive transfusion of blood products [42]. In select cohorts, such as pediatric patients and those with traumatic brain injury, LTOWB has been associated with improved outcomes [26–28, 43]. Over the past decade, there has been a nationwide transition at many trauma centers to transfusion of LTOWB as the product of choice for these resuscitative measures [26–28, 43]. 

Low-titer O negative whole blood has been safely administered in a single-center cohort study, with no transfusion reactions in the whole blood group [23]. Researchers at Barnes-Jewish Hospital performed a prospective observational trial over an eighteen-month period comparing outcomes before and after whole blood was introduced systematically. In this study of 86 patients, there was no unadjusted difference in survival, although there was an improved odds of survival in the whole blood group after adjusting for maximum clot firmness as measured by viscoelastic testing [26]. One retrospective cohort study of 270 matched patients indicated no difference in clinical outcomes [17]. A second large retrospective analysis of the data from the Trauma Quality Improvement Program indicated that 280 patients receiving whole blood had reduced 24-hour and hospital mortality, and a reduced rate of complications after adjustment for clinical covariates [22]. However, none of these studies were randomized, most used O-negative whole blood or a mixture of O-positive and O-negative whole blood, and there was no consideration of alloimmunization of female recipients against the Rh positive whole blood [34–37, 44]. One meta-analysis indicated a significant mortality reduction with whole blood use for traumatic hemorrhagic shock; a second meta-analysis performed by the Eastern Association for the Surgery of Trauma did not demonstrate mortality reduction but did show transfusion reduction [24, 45]. 

Two randomized controlled trials of whole blood have been performed for adults with traumatic hemorrhagic shock, but have recognized limitations. In 2013, Cotton et al. published a trial of 107 patients randomized to leukoreduced type specific whole blood versus component therapy (CT) for type O and type A recipients. In this trial, patients were blood typed prior to inclusion in the study. Due to the need to have typed product prior to transfusion, many patients were excluded at the discretion of the attending trauma surgeon as being too high-risk to wait for type-specific whole blood and received immediately-available CT. This study demonstrated a reduction in transfusion volumes for patients receiving whole blood after those with severe brain injury were excluded [27]. A second randomized study was conducted by Duschene et al. comparing whole blood transfusion to CT. The whole blood group had lower plasma and RBC utilization in the first 24 h, but higher rates of emergency department mortality [28]. However, this study had imbalances between cohorts with respect to mechanism of injury, demographics, and shock index. Other randomized controlled trials are currently proposed, but have not yet been executed [46, 47]. 

With the US Food and Drug Administration reporting rates of transfusion related acute lung injury (TRALI) and other blood transfusion reactions at rates of 1 in 5000 PRBC units and 1 in 2000 FFP units, efforts to decrease units of transfusion inherently minimize patient risk [48]. Given that the largest savings in PRBC and FFP transfusion occurred in patients undergoing HCI, one possible mechanism is reduced coagulopathy with LTOWB transfusion as compared to PRBC and FFP transfusion, as discussed extensively above. Additionally, the reduction in cryoprecipitate use with LTOWB compared with CT is a novel observation, and bears further study. Based on differences in fibrinogen activity in different plasma preparations, this finding might be due to increased fibrinogen activity in whole blood as compared to fresh frozen plasma [49]. The superior clotting ability of whole blood has been demonstrated out to 21 days with thromboelastography data [50]. Fibrinogen levels in cold stored whole blood are higher than in mixed components (RBC and lyophilized plasma) at both 1 day and 14 days. Prothrombin time is closer to normal in cold stored whole blood than in mixed components at 1 and 14 days. Similarly, cold stored whole blood has higher levels of Factors V and XIII at 1 and 14 days, and has higher levels of Factor VIII at 1 day. EXTEM clotting times were relatively similar in both cold stored whole blood and mixed components at 1 and 14 days, but the amount of clot formed after 5 min in the cold stored whole blood was much higher than for the mixed components at both 1 and 14 days, indicating superior platelet and/or fibrinogen function in the whole blood. In the presence of a platelet inhibitor, FIBTEM data indicates that the amount of clot formed after 5 min by cold stored whole blood is superior to mixed components at both 1 day and 14 days, indicating better fibrinogen function [51]. Thus there is a potential scientifically sound rationale for the treatment effect we demonstrated in our study.

Strengths of this study include a robust power analysis and pragmatic randomization scheme as well as good matching between treatment arms. The findings of transfusion reduction are highly significant, particularly for patients with HCI, and the resulting findings are novel for a RCT. These findings are bolstered by the within-groups comparison that indicates that CT patients with HCI utilized more PRBC, FFP, and PLT compared to their non-HCI counterparts; this was not true in the LTOWB group.

The findings may suggest that LTOWB should be preserved for patients undergoing HCI, which begs the question of how to select for these patients. Investigators from Grady Memorial Hospital identified transpelvic and transcavitary gunshot wounds, systolic blood pressure < 90 mm Hg, and base deficit greater than 10 mEq/L as risk factors for both massive transfusion and need for hemorrhage control [52]. Alternatively, if one accepts the premise that need for massive transfusion and need for hemorrhage control are well-correlated, established tools to predict the likelihood of massive transfusion such as the Assessment of Blood Consumption (two out of four risk factors including pulse > 120, systolic blood pressure < 90, penetrating mechanism, and positive focused abdominal sonographic assessment for trauma) or the Critical Administration Threshold (3 units PRBC transfused in 1 h) may be used for this purpose [53, 54]. While none of these measures are likely to be perfect, they may help isolate patients at higher risk for need of HCI, and thus more likely to benefit from LTOWB.

This study has several limitations. While overall survival did not differ between groups, the study was not powered to detect a mortality difference. However, both groups remained well matched for age, mechanism and degree of injury, and emergency department vital signs, unlike prior studies [28]. Because order for transfusion was ultimately at the discretion of treating trauma surgeon, bias in transfusion may have occurred. Regular audits of initiation and timeliness (within 10 min of onset of hypotension) of transfusion in hypotensive trauma patients were performed and compliance ranged from 84 to 92% over the past 12 months. No patterns of omission of transfusion or significant delays were identified. The pragmatic randomization scheme with 24-hour block assignment could be considered a limitation. Because a traditional block randomization schedule was not feasible in the time-pressured environment of an acute trauma resuscitation, the alternating 24-hour block scheme was utilized. The exclusion of transfusion data from patients who expired within 24 h also potentially limits applicability of the results, but was done to avoid survivor bias.

Because only male patients were included, broad applicability of the study findings are limited. As LTOWB has low levels of anti-A and anti-B titers, the risk of hemolysis should be minimal [17]. Likewise, previous data indicates that approximately 7.8% of Rh(D) negative trauma patients receiving uncrossmatched type O blood developed anti-D antibodies after approximately six months [35]. The rate of acute Rh alloimmunization was very low in a single-center study involving more than 5000 transfusions, with only one acute seroconversion [37]. In a large meta-analysis of more than 60,000 transfusions with nineteen inadvertent transfusions against clinically significant alloantibodies, only one case of anti-D reaction with hemolysis occurred. Thus empiric uncrossmatched transfusion is exceptionally safe [36]. Alloimmunization with anti-D has been prevented with Rh immunoglobulin, although use of this protective intervention is likely unfeasible during a massive transfusion protocol [44]. As there is increasing emphasis in the transfusion medicine field that the risk of death from hemorrhagic shock should outweigh the perceived risk of alloimmunization, expansion of LTOWB for initial resuscitation in patients excluded from the current study populations form the basis for future investigation.

The most notable limitation was that supply shortages of LTOWB throughout the course of this single institution study led to imbalanced accrual in the LTOWB and CT arms and potentially reduction in the potential effect size. Lack of availability of LTOWB was anticipated in this study and crossover from LTOWB to CT arms was allowed; intention to treat analysis was not performed. The reasons for lack of availability of LTOWB were multifactorial and include the limited supply of type O donors, increasing demand for type O blood products in non-trauma settings, requirements to mitigate transfusion-associated acute lung injury, and exclusion of blood from donors who use aspirin. Furthermore, although demand for LTOWB has increased, it is still understandably dwarfed by the demand for type O component units. This limitation constituted a random, not systematic, source of error in the trial [29, 30]. These supply limitations make both the study of LTOWB for effectiveness and the treatment of patients with LTOWB extremely challenging; the results of initial pragmatic studies such as this one may eventually prompt a sustained effort by trauma surgeons to advocate for increased LTOWB supply and thus change the supply curve to allow more patients to benefit from this treatment.

Historically, lessons learned from successive military conflicts have informed current transfusion protocols. Approximately 20% of military casualties require blood, and the amount of whole blood per casualty in the most recent conflicts has ranged from 6.8 to 13.3 units [55]. While a “walking blood bank” is not available in civilian settings, the development of systems that facilitate emergent blood transfusion, including whole blood, is of vital importance. Evolving techniques, such as increasing the permissible filtration window for whole blood from eight to twelve hours, may help improve whole blood supply [56]. Studies like this one that demonstrate overall transfusion savings with initial whole blood resuscitation provide evidence to support continued innovations in the supply chain to improve long-term availability of whole blood.

## Conclusion

This pragmatic RCT has demonstrated that index use of LTOWB is a viable alternative to CT in the frequent trauma setting of hemorrhagic shock in male patients, and may lead to decreased transfusion. Although LTOWB did not decrease all-cause mortality, use of LTOWB was associated with notably reduced transfusion of PRBC in patients undergoing HCI, and a trend toward reduction in transfusion of PRBC, FFP, and CRYO in all patients surviving 24 h. The current pragmatic trial design lends itself to future multicenter trials better powered for mortality endpoints and evaluation of RH alloimmunization issues in expanded patient populations. Further work will be required to improve access to LTOWB. When available, LTOWB should be considered as a primary source of volume based resuscitation in the adult male trauma population.

## Data Availability

No datasets were generated or analysed during the current study. Authors AMS and KM had full access to all the data in the study and take responsibility for the integrity of the data and the accuracy of the data analysis.
